# Identification of MRS2 Gene Family and Expression Analysis in Response to Magnesium Treatment in *Malus domestica*

**DOI:** 10.3390/plants14111672

**Published:** 2025-05-30

**Authors:** Jiying Bao, Huimin Gou, Shangwen Yang, Guoping Liang, Juan Mao

**Affiliations:** The College of Horticulture, Gansu Agricultural University, Lanzhou 730070, China; 18893954743@163.com (J.B.); ghm1648885861@163.com (H.G.); 155596765903@163.com (S.Y.); lianggp@gsau.edu.cn (G.L.)

**Keywords:** apple, CorA/MRS2 gene family, magnesium ion, expression analysis

## Abstract

The CorA/MRS2-type transporters represent a crucial family of magnesium ion transporters widely distributed in plants. Through comprehensive screening and alignment using the Phytozome database, we identified seven magnesium-related *MdMRS2* Confirm the deletion of the “Chinese Province” column in the address. genes in apple (*MdMRS2-1* to *MdMRS2-7*), which were distributed across seven distinct chromosomes. Phylogenetic analysis classified these genes into five distinct clades. Tissue-specific expression profiles revealed the differential expression patterns of *MdMRS2* members in different tissues such as the apple roots, stems, leaves, seedlings, seeds, flowers, and fruits. Among them, the expression level of *MdMRS2-5* was the highest in fruits, while that of *MdMRS2-6* was the lowest in seeds. Analysis of *cis*-regulatory elements in *MdMRS2* promoter regions identified numerous light-responsive elements, MYB binding sites, and hormone-responsive elements, suggesting their transcriptional regulation may be influenced by related metabolic pathways or signaling molecules. qRT-PCR results showed that the relative expression levels of all genes were significantly upregulated compared with CK under M3 treatment, while there were no significant differences in other treatments. Among them, the upregulation of *MdMRS2-7* was the most significant, increasing by 142% compared with CK. Notably, all *MdMRS2* genes were significantly upregulated under 4 mmol·L^−1^ MgSO_4_ treatment. Subcellular localization experiments conducted in tobacco leaves confirmed the membrane and cytoplasmic distribution of these transporters, consistent with bioinformatic predictions. These genes may become candidate genes for subsequent functional studies. This work will provide a basis for future research on the response mechanism and function of the MRS2 gene family in response to magnesium stress.

## 1. Introduction

Apple (*Malus domestica*), a perennial woody plant, is one of the most widely cultivated fruit trees worldwide, with China ranking first in both planting area and production [1]. This economically significant crop is highly valued by consumers for its pleasant sweet-sour taste and rich nutritional content [2].

Magnesium ions (Mg^2+^) serve as an essential element for plant growth and development, being a vital component of both chlorophyll and various enzymes [3,4]. In apple trees, magnesium deficiency not only impairs tree growth and development but also adversely affects fruit quality and yield. Numerous studies have demonstrated that magnesium deficiency in plants leads to leaf chlorosis, resulting in reduced chlorophyll content and decreased photosynthetic rate [5]. Furthermore, as the most abundant free divalent cation in plant cells, Mg^2+^ plays crucial roles in membrane stabilization, ion balance maintenance, and root growth [6]. Magnesium exists in plant systems predominantly as Mg^2+^ ions [7]. Beyond its crucial function in facilitating carbon assimilation during photosynthesis, Mg^2+^ serves three key physiological roles in chloroplasts—modulation of excitation energy distribution between photosystems II and I, participation in grana stacking processes, and contribution to thylakoid membrane assembly—collectively maintaining chloroplast structural integrity. Furthermore, while essential for photosynthetic efficiency and root absorption, Mg^2+^ dynamics exhibit additional complexity through its bidirectional transport within vascular systems, involving both xylem and phloem networks [8].

Magnesium ions (Mg^2+^) play vital roles in biochemical metabolism, and their selective permeability across biological membranes necessitates specialized transport systems. The uptake and translocation of Mg^2+^ are mediated by complex membrane protein systems, primarily through active transport mechanisms [9]. Current research has identified multiple Mg^2+^ transport systems in plants, including the cobalt resistance A protein family (CorA), Mg^2+^/H^+^ exchangers (*Arabidopsis thaliana* magnesium-proton exchanger, *AtMHX*), P-type phosphatases, the MgtE transporter family, and various ion channels [10]. Of these, CorA/MRS2-type transporters are particularly crucial for maintaining Mg^2+^ homeostasis in plants [11].

Following the completion of *Arabidopsis thaliana* genome sequencing, researchers identified the AtMRS2/AtMGT family in 2000, showing both structural and sequence homology to bacterial CorA proteins [12]. CorA-type homologs are evolutionarily conserved across all domains of life, and are present in archaea, eubacteria, and eukaryotes [13]. Bacterial systems employ three coordinated Mg^2+^ transporters, CorA, MgtA, and MgtB, with CorA demonstrating the highest Mg^2+^ affinity and serving as the primary membrane-localized transporter in prokaryotes [14]. The conserved GMN motif in CorA proteins has been experimentally confirmed as essential for Mg^2+^ transport functionality [14].

The *MRS2* gene, a eukaryotic homolog of bacterial CorA, was first characterized in yeast, where it encodes the Mrs2p protein—an integral component of the inner mitochondrial membrane [15] Subsequent research has revealed that CorA/MRS2-type magnesium transporters are widely distributed across plant species. In Arabidopsis thaliana, genomic studies have identified 11 family members, comprising 10 functional CorA/MRS2-type magnesium transporters and 1 pseudogene (*AtMRS2-9*) [16] Functional analyses in Arabidopsis have demonstrated that these transporters exhibit broad substrate specificity capable of mediating not only Mg^2+^ but also other divalent cations, including Fe^2+^ [14]

Among these, the *AtMRS2-1* gene represents one of the best-characterized members. This plasma membrane-localized transporter plays a crucial role in root magnesium acquisition, displaying both high affinity and specificity for Mg^2+^ under physiological soil concentrations. While capable of transporting other divalent cations, this activity requires concentrations significantly exceeding typical soil levels [14].

In rice (*Oryza sativa*), nine CorA/MRS2-type magnesium transporter proteins have been identified. Among these, *OsMGT1* demonstrates plasma membrane localization and exhibits significant alleviation effects on aluminum toxicity in rice roots [17]. This gene shows predominant expression in shoots and callus tissues [18]. Similarly, six CorA/MRS2-type magnesium transporter proteins were identified in grapevine (*Vitis vinifera*), with predicted involvement in chloroplast metabolism [8].

Multiple CorA/MRS2-type Mg^2+^ transporters have also been characterized in various horticultural crops, including tobacco (*Nicotiana tabacum*) and peach (*Prunus persica*). These transporters collectively regulate root Mg^2+^ uptake and enhance plant adaptation to Mg^2+^-deficient environments [19]. Specifically, seven MRS2/MGT family members were identified in the tobacco genome. *NtMGT1* shows root-specific expression, while *NtMGT2*, *NtMGT4*, and *NtMGT5* display leaf-specific expression with light-inducible characteristics [20].

The presence of multiple magnesium stressors in the natural environment poses a serious threat to plant growth and development, thereby limiting the sustainable development of agriculture [21]. Magnesium ion transporters can reduce the toxicity of aluminum (Al) in plants. *AtMRS2-1,* located on the vacuole, is insensitive to Al stress, while *AtMRS2-10* and *AtMRS2-11* show high sensitivity to Al toxicity [22]. It also has an impact on the development of pollen. *PbrMGT7* was also expressed in the pollen, and acted on the mitochondria to keep the homeostasis of Mg^2+^ in pollen development [23]. Although it is known that a large amount of research has been conducted on magnesium ion transporters in other species, as far as we know, they have not yet been studied in apples. Therefore, an integrated approach was adopted in this study: the *MdMRS2* gene was identified through apple genome screening, and systematic bioinformatics analysis was performed to reveal the function of the *MdMRS2* gene in magnesium ion transport. The experimental treatments under magnesium-deficient and over-magnesium conditions were designed using apple isolate seedlings to determine the expression pattern of the *MdMRS2* gene in response to different magnesium concentrations to provide targets for the subsequent genetics, to verify its role in apple magnesium utilization efficiency and stress tolerance, and to improve the quality of apple seedlings by optimizing magnesium fertilizer application strategies and combining key gene expression patterns.

## 2. Results

### 2.1. Physicochemical Properties Analysis of the MdMRS2 Gene

**Figure 1:**
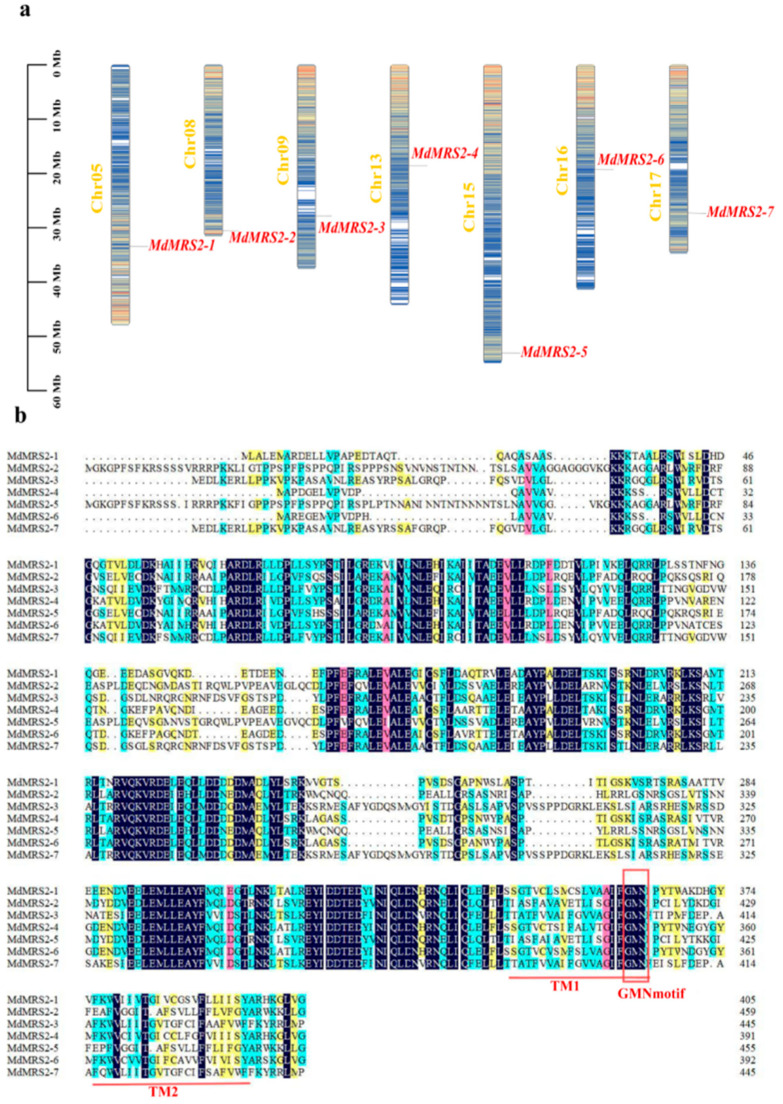
Chromosomal distribution and multiple sequence alignment analysis of MRS2 gene family members in apple. (**a**) Chromosome localization. The left scale indicates the chromosome length (Mb). (**b**) Multiple sequence alignment. The two red lines labeled are the two structural domains, TM1 and TM2. The red box labeled is the GMN motif.

Through HMMER model-based homology analysis, seven apple *MRS2* genes were identified and designated as *MdMRS2-1* through *MdMRS2-7* (Table 1). Chromosomal localization analysis revealed that these MRS2 family members were distributed across seven distinct apple chromosomes. Specifically, *MdMRS2-1* was mapped to chromosome 5, *MdMRS2-2* to chromosome 8, *MdMRS2-3* to chromosome 9, *MdMRS2-4* to chromosome 13, *MdMRS2-5* to chromosome 15, *MdMRS2-6* to chromosome 16, and *MdMRS2-7* to chromosome 17 (Figure 1a), demonstrating an even distribution throughout the apple genome.

Analysis indicated that the amino acid content of the MdMRS2 protein ranged from 392 (MdMRS2-4) to 460 (MdMRS2-2), and the relative molecular mass ranged from 43724.98 (MdMRS2-4) to 51343.86 (MdMRS2-2) Da (Table 1). The isoelectric point ranges from 4.74 (MddMRS2-1) to 8.22 (MdMRS2-5). Among them, the theoretical isoelectric point of MdMRS2-5 is greater than 7, and it is a basic amino acid. The other six genes are all less than seven and are acidic amino acids. Subcellular localization prediction indicates that the MdMRS2 gene is located in different sites, including the nucleus, cell membrane, chloroplast, and cytoplasm.

Multiple sequence alignment of the seven apple MRS2 protein sequences revealed that all MdMRS2 proteins exhibited the characteristic Gly-Met-Asn (GMN) tripeptide motif at their C-termini—a hallmark feature of magnesium transporters—along with two distinct transmembrane domains (TMD1 and TMD2) (Figure 1b).

### 2.2. Phylogenetic Analysis of MRS2 Gene Family

**Figure 2:**
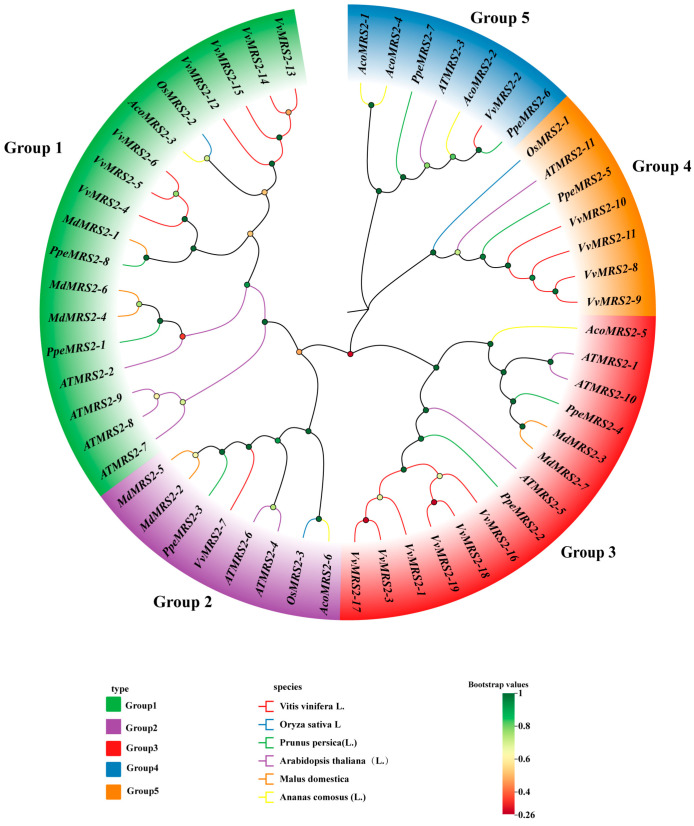
Phylogenetic analysis of *MRS2* gene in *Malus domestica*, *Oryza sativa*, *Arabidopsis thaliana*, *Vitis vinifera*, *Ananas comosus*, and *Prunus persica* of *MdMRS2.* Group 1 to Group 5 are divided into five subgroups. Squares of different colors represent different subgroups. Green represents Group 1, purple represents Group 2, red represents Group 3, blue represents Group 4, and orange represents Group 5. Lines of different colors represent different species: red represents grapes, blue represents rice, green represents peaches, purple represents Arabidopsis, orange represents apples, and yellow represents pineapples. The dots in the figure represent the self-expanding value. The redder the color, the larger the self-expanding value and the closer the genetic relationship. The greener the color, the smaller the self-expansion value and the farther the genetic relationship.

To elucidate the functional characteristics of the MRS2 gene family, we performed multiple sequence alignment of seven identified MdMRS2 proteins along with known *MRS2* homologs from *Oryza sativa*, *Arabidopsis thaliana*, *Vitis vinifera*, *Ananas comosus*, and *Prunus persica*. A maximum parsimony phylogenetic tree was constructed (Figure 2), revealing five distinct subfamilies.

Among these, the seven apple *MRS2* genes were distributed across three subfamilies: subfamily Group 1 (*MdMRS2-1*, *MdMRS2-4*, *MdMRS2-6*), Group 2 (*MdMRS2-2*, *MdMRS2-5*), and Group 3 (*MdMRS2-3*, *MdMRS2-7*), while no apple *MRS2* members were identified in the subfamilies Group 4 or Group 5. It is worth noting that phylogenetic analysis indicates that the *MdMRS2* gene has a closer evolutionary relationship with dicotyledonous plants than with monocotyledonous plants. It indicates a closer genetic relationship with dicotyledonous plants.

### 2.3. Synteny Analysis and Selection Pressure Analysis

**Figure 3:**
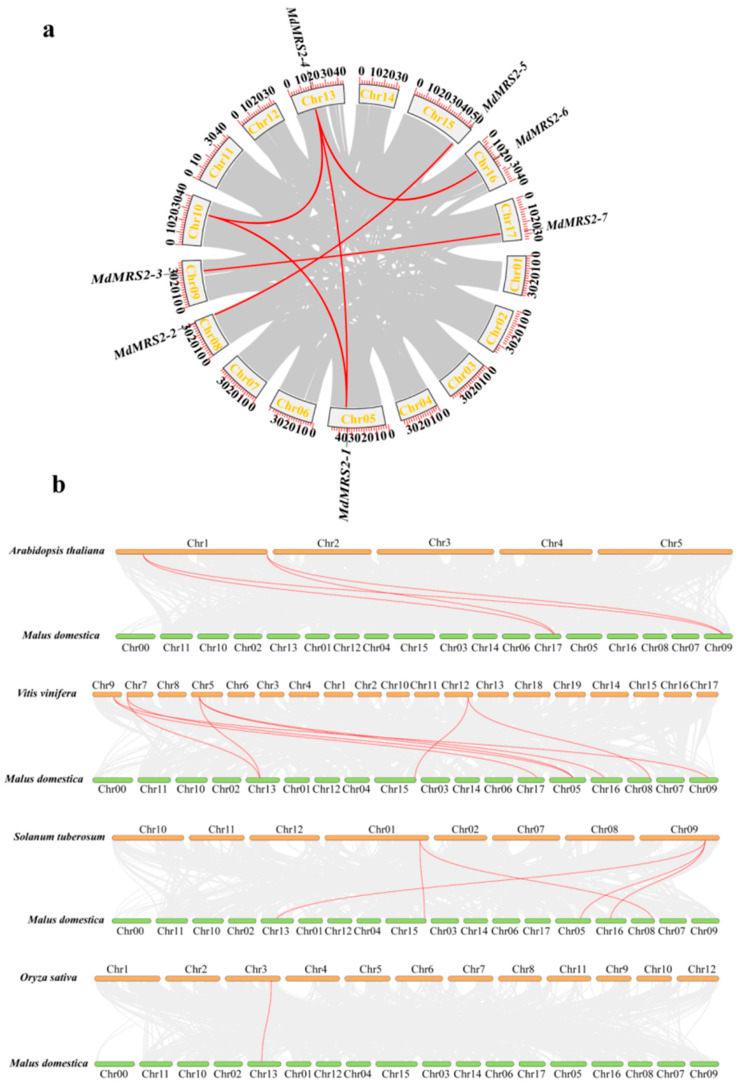
Collinearity analysis. (**a**) Intraspecific collinearity analysis. (**b**) Interspecific collinearity analysis. The red lines represent duplicate pairs of *MdMRS2* genes.

Collinear genes represent paralogous genes that may exist in another genome through duplication while maintaining identical sequential order, whereas paralogous genes specifically originate from gene duplication events occurring after species formation. Synteny analysis was conducted using TBtools software v2.210 to investigate the duplication events of apple *MRS2* genes. The results demonstrated (Figure 3a) four distinct pairs of collinear genes involved in segmental duplication, *MdMRS2-2/MdMRS2-5*, *MdMRS2-4/MdMRS2-6*, *MdMRS2-3/MdMRS2-7*, and *MdMRS2-1/MdMRS2-4*, showing that significant gene duplication events existed in the apple MRS2 gene family.

Interspecies synteny analysis between apple (*Malus domestica*) and four representative species, *Arabidopsis thalian*, *Vitis vinifera*, *Oryza sativa*, and potato (*Solanum tuberosum),* revealed 4, 9, 5, and 1 syntenic gene pairs, respectively (Figure 3b). It is worth noting that, compared with dicotyledonous plants, apples have a closer genetic relationship with monocotyledonous plants.

Ka/Ks analysis of apple *MRS2* genes (Table 2) revealed that four homologous gene pairs exhibited Ka values in a range of 0.02~0.17 and Ks values in a range of 0.16~1.95. All *MRS2* gene pairs showed Ka/Ks ratios significantly less than 1, indicating that the nonsynonymous substitution rate was substantially lower than the synonymous substitution rate. These results suggest that the *MRS2* gene family members have predominantly undergone purifying selection during evolution.

### 2.4. Secondary and Tertiary Structure Analysis of Apple MRS2 Protein

**Figure 4:**
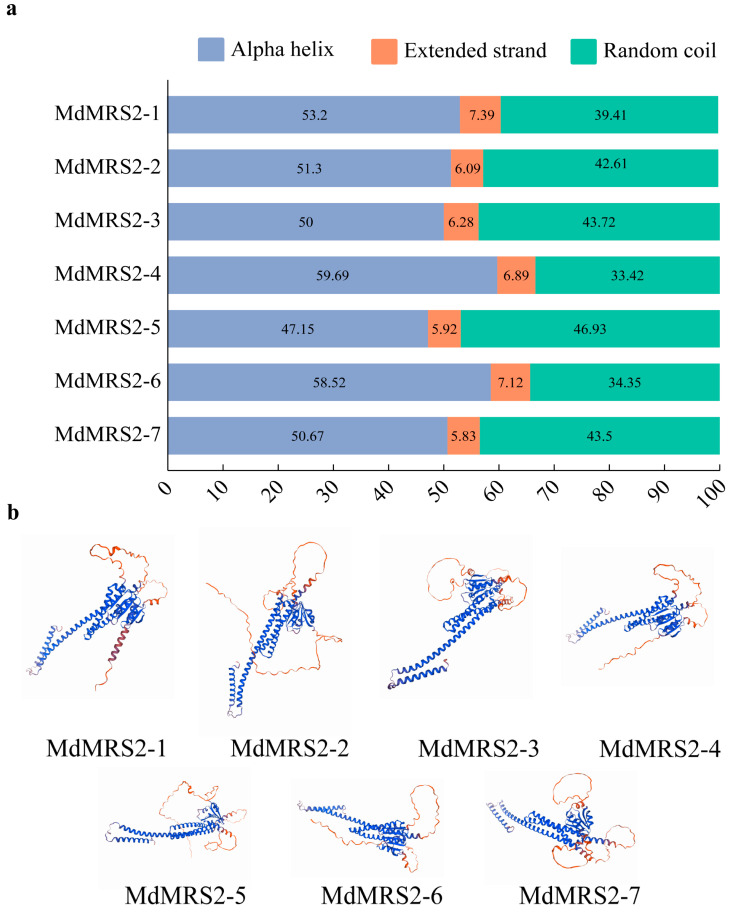
Analysis of the secondary and tertiary structures of the apple MRS2 gene family. (**a**) Secondary structure of the MdMRS2 gene family: purple for Alpha helix, orange for Extender chain, green for Unevenly curled. (**b**) Tertiary structure analysis of the MdMRS2 gene family in apple.

In order to further study the structural characteristics of the MdMRS2 protein, we conducted a secondary structure analysis on it. The results show that all family members are composed of α-helices, extension chains, and random coils (Figure 4a). It is notable that none of the seven MRS2 proteins contains a β-turn. The proportion of α-helical structures was the highest (47.15–59.69%), among which MdMRS2-4 had the largest proportion and MdMRS2-5 had the smallest proportion. The random coil was the second most abundant structure (33.42–46.93%), with MdMRS2-5 accounting for the largest proportion and the extended coil having the smallest proportion, which was only 5.83–7.39% (Figure 4b).

### 2.5. Gene Structures, Motifs, and Cis-Acting Elements of MdMRS2 Genes

**Figure 5:**
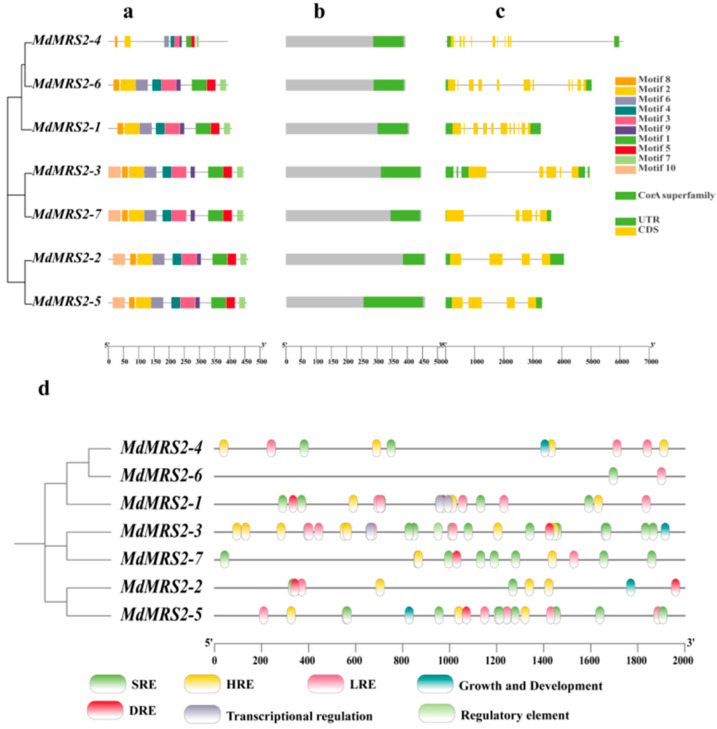
The structure, conserved domain, motif, and *cis*-acting elements of *MdMRS2* proteins. (**a**) Motif analysis of MdMRS2 proteins. (**b**) Domain analysis of MdMRS2 proteins. (**c**) Structure of *MdMRS2* genes. (**d**) *Cis*-acting element analysis.

Conserved motif analysis of MRS2 gene family members was performed using the MEME Version 5.5.8 online software, identifying 10 characteristic motifs (Figure 5a). Results revealed that *MdMRS2-1*, *MdMRS2-4,* and *MdMRS2-6* lacked motif 10, while the remaining four MdMRS2 proteins contained all ten motifs and displayed conserved structural organization in their motif arrangements. Structural domain analysis of the apple MRS2 gene family demonstrated that all seven apple MRS2 gene family members possessed characteristic domains of the CorA superfamily (Figure 5b).

Gene structure analysis of the seven *MdMRS2* genes confirmed that each contained complete gene features, including exons, introns, and both 5′ and 3′ untranslated regions (UTRs) (Figure 5c). Specifically, *MdMRS2-2* and *MdMRS2-5* exhibited three introns and four exons, while *MdMRS2-4* and *MdMRS2-6* contained ten introns and eleven exons. All seven *MdMRS2* genes maintained more than four exons in their coding sequences and preserved the complete gene structure with introns and flanking non-coding regions.

To further elucidate the potential functions of the apple MRS2 gene family, we analyzed the 2 kb upstream sequences (2000 bp before the start codon) for *cis*-acting elements. The results demonstrated that the apple MRS2 gene family contains numerous promoter-associated elements (Figure 5d). All seven *MdMRS2* genes possessed not only core promoter elements (TATA-box) and enhancer elements (CAAT-box), but also various other *cis*-acting elements, including those involved in stress response, transcriptional regulation, circadian rhythm, hormone response, and growth/development regulation.

### 2.6. Codon Bias Analysis of MdMRS2 Gene Family

**Figure 6:**
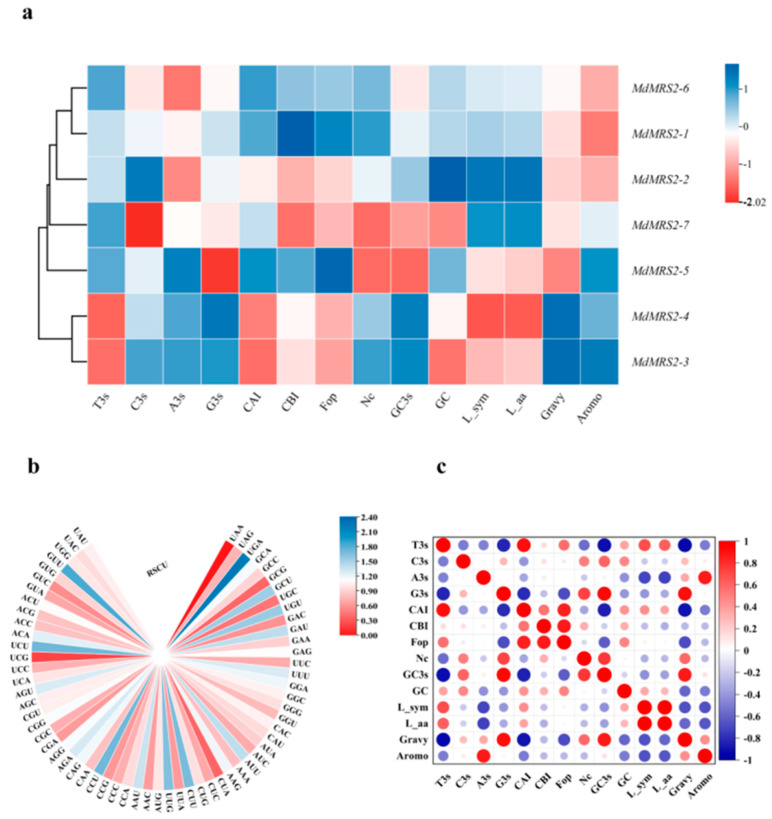
Codon preference analysis of the MRS2 family in apple. (**a**) Synonymous codon preference and correlation analyses of the *MdMRS2* gene. (**b**) The heat map of the RSCU for the *MdMRS2* gene. (**c**) Codon usage indexes correlation analysis of the *MdMRS2* gene. Blue color represents negative correlation, red color represents positive correlation, and white color represents no correlation. T3s indicates the amount of the third T of codons in amino acids containing synonymous codons ending in T. C3s indicates the amount of the third C of codons in amino acids containing synonymous codons ending in C. CBI represents the codon bias index. Nc represents the number of active codons. GC3s represents the frequency of G or C in the third base of the codon. GC represents the amount of G and C in the gene. L-sym represents the number of synonymous codons. L-aa is the total number of amino acids. Gravy is the average value of hydration, and Aromo is the motility of the protein.

In the deviation of synonymous codon usage, *MdMRS2-1* exhibited the highest Nc value of 56.81, whereas *MdMRS2-5* showed the lowest Nc value of 52.67 (Figure 6a). The RSCU values for seven coding sequences (CDSs) of the apple MRS2 gene family were calculated (Figure 6b). Analysis revealed 16 codons terminating with A, G, C, or U in apple *MRS2* genes. Among these, 30 codons showed high-frequency usage (RSCU > 1), with the following distribution: 14 ending with U, 8 with G, 5 with A, and 3 with C. Notably, the UGA codon exhibited the strongest preference (RSCU > 2), while all other high-frequency codons displayed moderate preference (RSCU < 2.0). Interestingly, GUU (Val) represented the most frequently used codon (RSCU = 1.84), whereas UCG (Ser) was the least frequent (RSCU = 0.21). Correlation analysis of codon usage patterns in the apple MRS2 family (Figure 6c) demonstrated significant relationships: T3s showed negative correlations with G3s, GC3s, and Gravy; G3s correlated negatively with CAI and Fop; Fop (frequency of optimal codons) exhibited positive correlations with CAI (codon adaptation index) and CBI (codon bias index), while G3s was positively correlated with GC3s and Gravy.

### 2.7. Tissue-Specific and qRT-PCR Analysis of Apple MRS2 Gene Family

**Figure 7:**
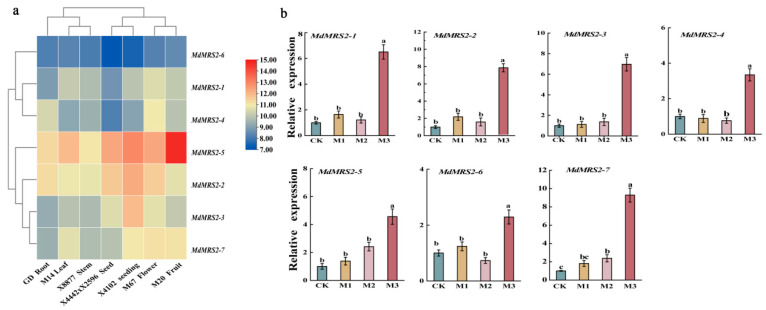
Tissue-specific and qRT-PCR analysis. (**a**) Tissue expression analysis of apple *MRS2* genes. Heat map experiments were performed using Gene Chip microarrays from the apple tissue expression database (GEO: GSE42873). Red represents high gene expression, and blue represents low gene expression. Flower data were M67, fruit data were M20, leaf data were M14, root data were GD, stem data were X8877, seed data were 4442 × 2596, and seedling data were X4102. (**b**) Analysis of *MdMRS2* family expression under different treatments. Three replicates were set for each treatment. Different lowercase letters indicate a significant difference at the 0.05 level, and the same lowercase letter indicates no statistical difference (*p* < 0.05).

The expression patterns of seven *MdMRS2* genes were comparatively analyzed by examining their tissue-specific expression profiles. As shown in Figure 7a, all seven genes have certain expression levels. Among them, the expression levels of *MdMRS2-2*, *MdMRS2-3*, *MdMRS2-5*, and *MdMRS2-7* were relatively high, while those of *MdMRS2-1*, *MdMRS2-4*, and *MdMRS2-6* were relatively low. Among them, the expression level of MdMRS2-5 was the highest in fruits, and that of *MdMRS2-6* was the lowest in seeds.

The relative expression levels of *MRS2* genes were analyzed following 48 h induction treatments with CK (1 mM MgSO_4_), M1 (0 mM MgSO_4_), M2 (2 mM MgSO_4_), and M3 (4 mM MgSO_4_). The results revealed differential expression patterns of *MRS2* genes across the treatment conditions (Figure 7b). The qRT-PCR results showed that the relative expression levels of all genes were significantly upregulated compared with CK under M3 treatment, while there were no significant differences in other treatments. Among them, the upregulation of MdMRS2-7 was the most significant, increasing by 142% compared with CK.

### 2.8. Protein Interaction Analysis

To investigate potential interactions among proteins encoded by the apple MRS2 genes, we constructed a protein interaction network. The results (Figure S1) showed that there was no interaction among MRS2 proteins, and MRS2-4 showed complete isolation with no detectable interactions. Other MRS2 members exhibited distinct interaction profiles with six partner proteins. Interaction analysis predicted functional associations between most apple MRS2 proteins and magnesium transporters (DVH24_005790 and DVH24_038971), indicating their cooperative involvement in plant magnesium ion transport processes.

### 2.9. Subcellular Localization of MdMRS2-3

**Figure 8:**
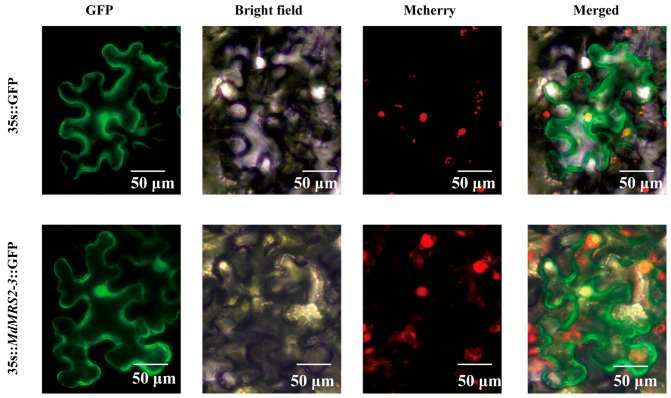
Subcellular localization of *MdMRS2-3*. The upper row shows the positive control (35S:: GFP), and the lower row shows the 35S::*MdMRS2-3*::GFP construct. The scale is 50 μm. Green fluorescent protein (GFP) and bright field (BF) were demonstrated.

The 35S::MdMRS2-3::GFP construct was introduced into the lower epidermal cells of wild-type tobacco leaves containing nuclear-localized mCherry via Agrobacterium-mediated transformation for transient expression analysis. Results showed that control cells transformed with empty 35S::GFP exhibited diffuse green fluorescence throughout the cell, while tobacco leaves expressing 35S::*MdMRS2-3*::GFP displayed fluorescence signals localized to both the plasma membrane and nucleus, with GFP fluorescence overlapping with nuclear Mcherry to produce yellow emission. This confirms that *MdMRS2-3* localizes to both the nucleus and plasma membrane (Figure 8), consistent with the predicted subcellular localization pattern.

## 3. Discussion

The MRS2 gene family is a typical magnesium transporter [24]. Plant magnesium transporters play an important role in the absorption and transport of magnesium in plants [25]. The identification and cloning of the MRS2 gene family have been reported in many plants. In this study, seven apple *MRS2* genes on different chromosomes were screened by bioinformatics methods and named as *MdMRS2-1* ~ *MdMRS2-7,* according to the chromosome position. These members all contain a transmembrane structure and a conserved GMN characteristic motif. In this motif, G is necessary for magnesium uptake, M maintains the integrity of the ion channel conformation, and N stabilizes these two functions [26]. In this paper, the evolutionary relationship of the *MRS2* system of six species was analyzed. The results show that the MRS2 gene family has five subfamilies, among which *MRS2* has the closest genetic relationship with dicotyledonous plants of the Rosaceae family, which is similar to the research results of Zhao et al. [22]. And the same branch may have similar functions. For example, the *AtMRS2-7* gene and the *MdMRS2-1*, *MdMRS2-4*, and *MdMRS2-6* sequences belong to the same branch. In *Arabidopsis thaliana*, the *AtMRS2-7* gene is a key gene to ensuring the survival of Arabidopsis thaliana under low magnesium conditions [27]. Therefore, it is speculated that *MdMRS2-1*, *MdMRS2-4,* and *MdMRS2-6* genes in apple have similar functions to ensure the survival of plants under low magnesium conditions. In phylogenetic tree and collinearity analysis, it was found that the closeness between apples and dicotyledonous plants is greater than that between monocotyledonous plants. The relationship between tomatoes (dicotyledonous plants) and *Arabidopsis thaliana* is closer than that between tomatoes and rice. However, Tong Mengying [28] found in her research on MaMRS2 that bananas (monocotyledonous plants) have a relatively distant genetic relationship with Arabidopsis thaliana (dicotyledonous plants) but a relatively close relationship with the monocotyledonous plant rice; the above results can indicate that the similarity between the CorA/MRS2 genes in plants is species-related. Zhao et al. found in the study of the grape MRS2 family that the Ka/Ks of all members of the VvMRS2 family were significantly lower than 1 [8]. In this study, it was also found that the Ka/Ks of all members of the MdMRS2 family were also significantly lower than 1, indicating that the nonsynonymous substitution rate is much lower than the synonymous substitution rate. These results indicate that the members of the MRS2 gene family have mainly undergone purification selection during evolution.

In papaya, CpMGT is mainly distributed within membrane cells [29]; in corn ZmMGT and Brazilian rubber tree HbMGT are mainly distributed in chloroplasts [30,31]. Liu et al. found that the subcellular localization prediction of tomatoes showed that *SlMRS2-1*, *SlMRS2-5,* and *SlMRS2-11* were located on the plasma membrane; *SlMRS2-2* and *SlMRS2-3* were located on the chloroplast membrane; *SlMRS2-1* was located on the cytoplasm; and *SlMRS2-4* was located on the nucleus and plasma membrane [19]. Wang Yongjun’s research found that eight MGT members in sugarcane are located on chloroplasts, while the remaining two are predicted to be located on mitochondria (*SsMGT3*) and the plasma membrane (*SsMGT5*) [32]. In his research on the magnesium ion transporter protein of pineapple, Hu Bingyan made transient expression of the *AcoMRS2-3* gene and found that its localization in tobacco cells was in the Golgi apparatus, endoplasmic reticulum, and plastids [33]. In *MdMRS2*, *MdMRS2-1* is located in the nucleus and chloroplasts; *MdMRS2-2* is located on the cell membrane and in the nucleus; *MdMRS2-3* is located in the cell membrane, nucleus, and chloroplasts; *MdMRS2-4* is located in the cytoplasm and chloroplasts; *MdMRS2-5* is located in the chloroplasts; *MdMRS2-6* and *MdMRS2-7* are located in the cell nucleus. The transient transformation of the *MdMRS2-3* gene revealed that it is located in the nucleus, cytoplasm, and chloroplast of tobacco, indicating that *MdMRS2* is likely to maintain the dynamic balance of magnesium ions in tissue cells in a multi-member cooperative manner.

By analyzing the primary structure of MdMRS2 family proteins, a total of 10 conserved motifs were identified, among which motif 1, motif 2, motif 3, motif 4, motif 5, motif 6, motif 7, and motif 8 existed in all the protein members, and it was speculated that these conserved motifs were important for the function of MdMRS2 family proteins. Tan et al. found in MeMGT that the proteins of this family are mainly composed of α-helix, extended strand, β-turn, and random curl, among which the proportion of β-turn is the lowest, ranging from 1.21% to 4.26% [34]. In the analysis of the secondary and tertiary structures of the MdMRS2 family proteins, it was revealed that the secondary structures of the seven proteins predominantly consisted of α-helices, extended strands, and random coils. Notably, β-sheets were absent, which may potentially be attributed to natural selection or evolutionary pressures. Upon examining their tertiary structures, it was observed that all seven proteins exhibited highly similar spatial conformations.

The *cis*-acting elements of the promoter play an important role in the transcription level of the gene, and the analysis of the *cis*-acting elements provides a certain reference value for predicting gene function [35]. By analyzing the promoter sequence of the gene, its role in intracellular and intercellular signaling pathways can be predicted to determine its function in cells. The homeostatic elements in chili peppers include light reaction elements and anaerobic induction response elements, while other components are restricted to one or more members of the chili MGT. For example, circadian rhythm response elements, endosperm expression response elements, seed-specific regulatory elements, wound response elements, and hypoxia-specific induction elements are, respectively, present only in the promoter regions of *MGT3/10*, *MGT10*, *MGT9*, *MGT3*, and *MGT9* in chili peppers [8]. Wang et al. conducted an expression analysis of the MGT gene in the diurnal cycle of two species of sugar plants. *MGT9* and *MG10* were observed to have a peak expression in the middle of the night period in *S. spontaneum*, but they showed no diurnal expression in *S. officinarum*, indicating diurnal rhythms regulate these two *MGTs* in *S. spontaneum* rather than *S. officinarum* [32]. In this study, the promoter region of the MdMRS2 gene family was analyzed. The results showed that the promoter of the apple MdMRS2 gene family had *cis*-acting elements related to light response, hormone response, drought, and low-temperature response. Especially, the light response elements and gibberellin response elements appeared frequently; this indicates that the MdMRS2 gene family may play an important role in the growth and development of the apple. It plays an important role in the photosynthesis of plants by regulating the transport of magnesium ions by combining with light response elements. Three of the thirty translated amino acids of the MdMRS2 family have Ter, one of the triplet amino acids generated by a method with shortening, and all of their translated codons are termination codons. Codon bias analysis of the gene showed that T3s was negatively correlated with G3s, Gc3s, Gravy, G3s with CAI, and Fop. Fop was positively correlated with CAI and CBI, and G3s with GC3s and Gravy. (Figure 6) This suggests that the base type at position 3 of the synonymous codon influences the degree of codon usage preference.

The fluorescence quantitative results showed that under the conditions of Mg^2+^ deficiency and Mg^2+^ excess, the relative expression level would be significantly upregulated only under the condition of Mg^2+^ excess. Under other conditions, it was upregulated or downregulated to varying degrees compared with CK, but none were significant. This was consistent with the results for tomato [14]. Yan et al. [36] observed that the expression of the *MGT6* gene in Arabidopsis thaliana responded to low Mg^2+^ in root tissues and showed a corresponding response in high Mg^2+^, but the difference was not significant. When observing the phenotype of Arabidopsis thaliana under ultra-high Mg^2+^ conditions, it was found that there were serious phenotypic defects. These data suggested that the mgt6 mutant is not only compromised under low Mg^2+^ levels but also hypersensitive to high-Mg^2+^ stress. This gene family solves the problem of ionic toxicity by regulating magnesium ions. Therefore, we speculate that the regulation of magnesium ions in apples plays an important role in addressing ionic toxicity.

## 4. Materials and Methods

### 4.1. Materials and Treatments

The experiment was conducted in 2024 at the Laboratory of Fruit Tree Physiology and Biotechnology, College of Horticulture, Gansu Agricultural University. The plant materials consisted of in vitro-grown ‘Orin’ apple (*Malus domestica*) plantlets cultured on MS medium (4.42 g·L^−1^ MS basal salts, 30 g·L^−1^ sucrose, 6 g·L^−1^ agar, 0.1 mg·L^−1^ 6-Benzylaminopurine (6-BA), and 0.2 mg·L^−1^ Indole acetic acid (IAA), pH 5.8–6.0). The plants were maintained in a growth chamber under controlled conditions: 25 °C with 16 h light/20 °C with 8 h darkness.

After 40 days of culture, uniformly growing, healthy, and contamination-free plantlets were selected and transferred to a hydroponic system containing modified MS nutrient solution with varying magnesium concentrations. Four MgSO_4_ treatment groups were established: CK (control), 1 mmol·L^−1^ MgSO_4_; M1 (Mg^2+^-deficient), 0 mmol·L^−1^ MgSO_4_; M2 (moderate Mg^2+^), 2 mmol·L^−1^ MgSO_4_; M3 (high Mg^2+^), 4 mmol·L^−1^ MgSO_4_. Each treatment was replicated three times. Following 48 h of treatment, leaf samples were collected, immediately wrapped in aluminum foil, flash-frozen in liquid nitrogen, and stored at −80 °C for subsequent analysis.

### 4.2. Identification of the Apple MRS2 Gene Family

The conserved amino acid sequences of *MRS2* genes from Arabidopsis thaliana were employed as queries to identify putative apple MRS2 family members through BLAST homology searches on Phytozome13 (https://phytozome-next.jgi.doe.gov/ accessed on 15 August 2024). Candidate genes were initially screened for redundancy using DNAMAN software v6.0, followed by domain validation through HMMER online analysis (https://www.ebi.ac.uk/Tools/hmmer/ accessed on 15 August 2024) to confirm the presence of characteristic *MRS2* domains (PF01544.21). Physicochemical properties, including amino acid length, molecular weight, and isoelectric point, were determined using the Expasy ProtParam tool (https://web.expasy.org/protparam/ accessed on 19 August 2024). Subcellular localization predictions were performed using both WoLF PSORT (https://wolfpsort.hgc.jp/ accessed on 19 August 2024) and CELLO v.2.5 (http://cello.life.nctu.edu.tw/ accessed on 19 August 2024) bioinformatics platforms.

### 4.3. Phylogenetic Tree, Synteny Analysis

The MRS2 family members from Arabidopsis thaliana, apple (*Malus domestica*), grape (*Vitis vinifera*), rice (*Oryza sativa*), peach (*Prunus persica*), and pineapple (*Ananas comosus*) were identified through Phytozome v13 (https://phytozome-next.jgi.doe.gov/ accessed on 24 August 2024). The amino acid sequences of these *MRS2* genes were converted to FASTA format using ClustalX v1.83, followed by phylogenetic tree construction with MEGA 7.0 software. The resulting phylogenetic tree was visualized and annotated using TVBOT (https://www.chiplot.online/tvbot.html accessed on 24 August 2024).

The whole-genome sequences of Arabidopsis thaliana, apple (*Malus domestica*), grape (*Vitis vinifera*), rice (*Oryza sativa*), and potato (*Solanum tuberosum*) were obtained from Phytozome v13 (https://phytozome-next.jgi.doe.gov/) (accessed on 19 August 2024). Subsequently, synteny analysis was performed using TBtools to identify conserved genomic blocks and homologous gene pairs among these species.

### 4.4. Ka/Ks, Motif, Gene Structure, and Cis-Acting Element Analysis

Gene structure analyses were conducted using CD-search (https://www.ncbi.nlm.nih.gov/Structure/bwrpsb/bwrpsb.cgi accessed on 1 September 2024) for conserved domain identification. MEME Suite (https://meme-suite.org/meme/tools/meme accessed on 1 September 2024) was employed for protein motif analysis, while TBtools facilitated covariance analysis, chromosomal localization mapping, and selection pressure analysis. Protein secondary structure was predicted via ExPASy ProtParam (https://web.expasy.org/protparam/ accessed on 8 September 2024), and tertiary structure modeling was performed using DNAMAN with ESPript (https://espript.ibcp.fr/ESPript/cgi-bin/ESPript.cgi accessed on 13 September 2024) for structural visualization. For regulatory element analysis, PlantCARE or equivalent tools were used to predict *cis*-acting elements within 2 kb upstream promoter regions. Protein–protein interaction networks were inferred using STRING (https://string-db.org/ accessed on 13 September 2024).

### 4.5. Codon Bias Analysis

Codon usage bias analysis was performed using CodonW v1.4.4 (http://codonw.sourceforge.net, accessed on 26 September 2024). The resulting data were processed and organized using Microsoft Excel 2010 (Microsoft Corporation, Redmond, WA, USA). For statistical correlation analysis, the formatted data were imported into OriginPro 2021 (OriginLab Corporation). Relative synonymous codon usage (RSCU) analysis was conducted using RStudio 2022.07.2 (https://www.rstudio.com/products/rstudio/download/, accessed on 4 December 2024) with the seqinr package.

### 4.6. RNA Extraction and qRT-PCR of MdMRS2-3 Gene

Total RNA was extracted from apple tissues using a plant RNA extraction kit (Acres Biotechnology Co., Ltd., Zhejiang, China) following the manufacturer’s protocol. RNA quality was assessed by measuring absorbance ratios (OD260/280) and concentration using spectrophotometry, with integrity verified by 1.2% agarose gel electrophoresis. Qualified RNA samples were stored at −80 °C until use.

For qRT-PCR analysis, gene-specific primers for *MRS2* genes were designed (Table S1) and synthesized by Sangon Biotech (Shanghai, China) Co., Ltd. (https://www.sangon.com accessed on 4 November 2024). First-strand cDNA was synthesized from 1 μg total RNA using the PrimeScript RT reagent Kit (Perfect Real Time; Takara Bio Inc., Kusatsu, Japan). Quantitative PCR was performed using SYBR Premix Ex Taq II (TakaraBio Inc.) on a LightCycler 96 Real-Time PCR System (Roche Diagnostics, Switzerland) with the following cycling conditions: initial denaturation at 95 °C for 30 s followed by 40 cycles of 95 °C for 10 s, 60 °C for 30 s, and 72 °C for 30 s. All reactions were performed in triplicate technical replicates.

### 4.7. Tobacco Transient Transformation

The full-length coding sequence of *MdMRS2-3* was amplified by PCR using cDNA derived from ‘Gala’ apple seedlings as template. The purified PCR product was subsequently cloned into the pCAMBIA2300-GFP expression vector through restriction enzyme digestion and ligation. The recombinant plasmid was transformed into DH5α cells (TransGen Biotech, Beijing, China), with positive clones initially screened by colony PCR. Verified clones were sent to Sangon Biotech (Shanghai) for Sanger sequencing. Following sequence confirmation, the recombinant plasmid was extracted and transformed into Agrobacterium tumefaciens strain GV3101 via electroporation. Positive Agrobacterium transformants were selected for subsequent plant transformation experiments.

The recombinant Agrobacterium culture harboring the target gene was centrifuged at 5000× *g* for 10 min at 4 °C, and the bacterial pellet was collected. The cells were resuspended in infiltration buffer (50 mL ddH_2_O + 500 μL MES + 200 μL MgCl_2_ + 75 μL As) to an optical density (OD600) of 0.7–0.8. The bacterial suspension was pressure-infiltrated into the abaxial surface of 25-day-old Nicotiana benthamiana leaves using a 2 mL sterile syringe, with empty vector (pCAMBIA2300) serving as negative control. Infiltrated plants were maintained in darkness at 25 °C for 48 h followed by 24 h under normal illumination (16 h light/8 h dark).

The injected bacterial solution was cut into the leaves with a scalpel to obtain the bacterial solution, and the samples were collected by the laboratory. A 0.2 mm × 0.2 mm microscopic sample was cut with a scalpel from the leaf injected with the bacterial solution and placed under an AX10 fluorescence inverted microscope (ZEISS, Germany) for observation and photographing.

### 4.8. Data Statistics and Analysis

The experimental data were organized and categorized using Microsoft Excel 2010, and graphical representations were generated using Origin 2021. Statistical analyses, including analysis of variance (ANOVA) and multiple comparisons, were performed using SPSS Statistics 22.0. Duncan’s multiple range test was employed for post hoc analysis of significant differences at a significance level of *p* < 0.05.

## 5. Conclusions

In this study, it was found that seven *MdMRS2* genes were distributed on seven different chromosomes of apple. The analysis of physical and chemical properties showed that the seven *MdMRS2* genes had no β-turn structure and contained the same GMN motif. According to its evolutionary relationship, it can be divided into five subfamilies. The analysis of *cis*-acting elements of the promoter showed that the MdMRS2 gene contains many *cis*-acting elements that can respond to hormones and environmental signals, such as *cis*-acting elements related to light response, hormone response, drought response, and low-temperature response; especially, light response elements and gibberellin response elements appear frequently. The results of qRT-PCR showed that the expression levels of *MdMRS2-2*, *MdMRS2-3,* and *MdMRS2-7* were relatively high under magnesium stress, and these genes may become candidate genes for subsequent functional studies. This work will provide a basis for future research on the response mechanism and function of the MRS2 gene family in response to magnesium stress.

## Figures and Tables

**Table 1:** Analysis of physicochemical properties of the MRS2 gene family in apple.

Gene Accession No.	Gene Names	Chromosomes	Amino Acid	Molecular Weight (Da)	Isoelectric Point	Subcellular Localization
MD05G1204800	*MdMRS2-1*	5	406	45,324.54	4.74	Chloroplast Nucleus
MD08G1238500	*MdMRS2-2*	8	460	51,343.86	5.62	Cell membrane Nucleus
MD09G1225700	*MdMRS2-3*	9	446	50,519.01	5	Nucleus Cell membrane Chloroplast
MD13G1205200	*MdMRS2-4*	13	392	43,724.98	4.82	Chloroplast Cytoplasm
MD15G1430500	*MdMRS2-5*	15	456	51,315.08	8.22	Chloroplast
MD16G1207000	*MdMRS2-6*	16	393	43,800.07	4.77	Nucleus
MD17G1224500	*MdMRS2-7*	17	446	50,526.93	5.07	Nucleus

**Table 2:** Selection pressure analysis of *MdMRS2*.

Paralogous Pairs	Ka	Ks	Ka/Ks
*MdMRS2-4/MdMRS2-1*	0.17	1.95	0.09
*MdMRS2-4/MdMRS2-6*	0.05	0.16	0.29
*MdMRS2-3/MdMRS2-7*	0.02	0.21	0.09
*MdMRS2-2/MdMRS2-5*	0.04	0.17	0.25

## Data Availability

The original contributions presented in this study are included in the article. Further inquiries can be directed to the corresponding author.

## Supplementary Materials

The following supporting information can be downloaded at: https://www.mdpi.com/article/10.3390/plants14111672/s1, Figure S1: Protein interaction. Nodes represent proteins, and lines between nodes represent interactions between proteins, with different colors corresponding to different types of interactions; Table S1: Real-time fluorescence quantification and amplification primers for expression analysis of the apple MRS2 gene family.

## References

[1] Fang S. Identification of the Drought Resistance Function of the Apple Transcription Factor MhMYB4 and Analysis of Its Mechanism of Action. Master’s Thesis.

[2] Ma Y. (2023). Analysis of CDPK Gene Family and Functional Identification of MdCDPK24 Resistance to Anthracnose in Apples of ‘Hanfu’ Apple.

[3] Ahmed F.F., Morsy M.H. Response of Anna apple trees grown in the new reclaimed land to application of some nutrients and ascorbic acid. Proceedings of the 5th Arabian Horticulture Conference.

[4] Li B. (2020). Effects of Magnesium Application on C and N Absorption, Utilization, Yield and Quality of Apples. Master’s Thesis.

[5] An H., Zhang X. (2020). Research Progress on Magnesium Deficiency Stress in Plants. J. Anhui Agric. Sci..

[6] Li Y., Liu X., Zhuang W. (2000). Research Progress on Plant Magnesium Nutritional Physiology. J. Fujian Agric. Univ..

[7] Yu X., Liu X. (2023). Research progress on the effect of magnesium on plants and the application of magnesium fertilizer in agriculture and forestry. South China Agric..

[8] Zhao X., Wu H., Zhang H., Ma Y., Wen T. (2023). Identification and bioinformatics analysis of MRS2 gene family in grape. Mol. Plant Breed..

[9] Du J. (2021). Molecular Identification and Functional Analysis of Magnesium Ion Transporter Family in Capsicum. Master’s Thesis.

[10] Cong Y., Luo D., Chen K., Jiang L., Guo W. (2012). Progress in the study of biological magnesium ion transporters. J. Agric. Biotechnol..

[11] Hermans C., Conn S.J., Chen J., Xiao Q., Verbruggen N. (2013). An update on magnesium homeostasis mechanisms in plants. Met. Integr. Biometal Sci..

[12] Bui D.M., Gregan J., Jarosch E., Ragnini A., Schweyen R.J. (1999). The bacterial magnesium transporter CorA can functionally substitute for its putative homologue Mrs2p in the yeast inner mitochondrial membrane. J. Biol. Chem..

[13] Wu X. (2019). Study on the regulation of magnesium nutrition and circadian clock in Arabidopsis.

[14] Liu W., Khan S., Tong M., Hu H., Yin L., Huang J. (2023). Identification and Expression of the CorA/MRS2/ALR Type Magnesium Transporters in Tomato. Plants.

[15] Deng P. (2007). *Arabidopsis thaliana* Mg^2+^ Functional Study of AtMGT3 Gene in Transporter Gene Family. Master’s Thesis.

[16] Gebert M., Meschenmoser K., Svidová S., Weghuber J., Schweyen R., Eifler K., Lenz H., Weyand K., Knoop V. (2009). A root-expressed magnesium transporter of the MRS2/MGT gene family in *Arabidopsis thaliana* allows for growth in low-Mg^2+^ environments. Plant Cell.

[17] Saito T., Kobayashi N.I., Tanoi K., Iwata N., Suzuki H., Iwata R., Nakanishi T.M. (2013). Expression and functional analysis of the CorA-MRS2-ALR-type magnesium transporter family in rice. Plant Cell Physiol..

[18] Li L., Tutone A.F., Drummond R.S., Gardner R.C., Luan S. (2001). A novel family of magnesium transport genes in *Arabidopsis*. Plant Cell.

[19] Zhou P., Yan S., Guo R., Jin G. (2024). Identification and Expression Analysis of Peach Magnesium Ion Transporter MGT Gene Family. Acta Hortic. Sin..

[20] Wu T., Wei X., Feng C., Huang Y., Xu S., Qiu F., Zheng Y., Li W., He H. (2022). Identification and expression analysis of the MRS2/MGT gene family in tobacco. Chin. Agric. Sci. Bull..

[21] Chen L., Cai D., Zhang L., Song S., Luo X., Chen Y., Li J., Xu T., Mao D. (2021). Research Progress on Magnesium Ion Transport and Magnesium Stress Response Mechanism in Plants. Life Sci. Res..

[22] Ishijima S., Manabe Y., Shinkawa Y., Hotta A., Tokumasu A., Ida M., Sagami I. (2018). The homologous Arabidopsis MRS2/MGT/CorA-type Mg^2+^ channels, AtMRS2-10 and AtMRS2-1 exhibit different aluminum transport activity. Biochim. Biophys. Acta BBA Biomembr..

[23] Zhao Z., Wang P., Jiao H., Tang C., Liu X., Jing Y., Zhang S., Wu J. (2018). Phylogenetic and expression analysis of the magnesium transporter family in pear, and functional verification of PbrMGT7 in pear pollen. J. Hortic. Sci. Biotechnol..

[24] Li H., Du H., Huang K., Chen X., Liu T., Gao S., Liu H., Tang Q., Rong T., Zhang S. (2016). Identification, and functional and expression analyses of the CorA/MRS2/MGT-type magnesium transporter family in maize. Plant Cell Physiol..

[25] Waters B.M. (2011). Moving magnesium in plant cells. New Phytol..

[26] Palombo I., Daley D.O., Rapp M. (2013). Why is the GMN motif conserved in the CorA/Mrs2/Alr1 superfamily of magnesium transport proteins?. Biochemistry.

[27] Mao D.D., Tian L.F., Li L.G., Chen J., Deng P.Y., Li D.P., Luan S. (2008). AtMGT7: An *Arabidopsis* gene encoding a low-affinity magnesium transporter. J. Integr. Plant Biol..

[28] Tong M., Liu W., He H., Hu H., Ding Y., Li X., Huang J., Yin L. (2020). Identification and functional analysis of the CorA/MGT/MRS2-type magnesium transporter in banana. PLoS ONE.

[29] Xu Y., Zou Z., Guo J. (2022). Kong, H.; Zhu, G.; Guo, A. Cloning and Functional Analysis of Magnesium Ion Transporter Gene CpMGT1 in Papaya. Chin. J. Trop. Crops.

[30] Li H., Zhang S., Chen Q. (2018). Expression Analysis of ZmMGT12 Gene in Maize and Genetic Transformation in *Arabidopsis*. Mol. Plant Breed..

[31] Yang J., Qin Y., Fang Y., Tang C. (2016). Cloning and expression analysis of magnesium ion transporter protein gene HbMGT10 in Brazilian rubber tree. J. Trop. Crops.

[32] Wang Y. (2019). Family Identification of MGT Gene in Sugarcane and Transcriptome Dynamics Study of Magnesium Deficiency Stress. Master’s Thesis.

[33] Hu B. (2019). Identification, Expression and Functional Study of CorA-MRS2-ALR Magnesium Ion Transporters Family in Pineapple. Master’s Thesis.

[34] Tan B., Zhang Y., Zhang P., Wang Z., Ma Q. (2024). Identification and bioinformatics analysis of magnesium transporter family genes in cassava. Biotechnol. Bull..

[35] Yang J., Gong L., Xu T., Zhao S., Yang Z., Wu Y. (2024). Identification and Expression Analysis of TST Gene Family in Grape. J. Northwest Bot..

[36] Yan Y.W., Mao D.D., Yang L., Qi J.-L., Zhang X.-X., Tang Q.-L., Li Y.-P., Tang R.-J., Luan S. (2018). Magnesium transporter MGT6 plays an essential role in maintaining magnesium homeostasis and regulating high magnesium tolerance in *Arabidopsis*. Front. Plant Sci..

