# Supplementary material for: Identification of MRS2 Gene Family and Expression Analysis in Response to Magnesium Treatment in Malus domestica
Source: Plants (Basel). 2025 May 30;14(11):1672. doi: 10.3390/plants14111672 (PMC12157029; doi:10.3390/plants14111672)
Supplement: Supplementary file 1 [file plants-14-01672-s001.zip › Table S1.pdf]

Table s1. Real-time fluorescence quantification and amplification primers for expression analysis of the apple MRS2 gene family

Gene name	Forward primer (5' to 3') and reverse primer (5' to 3')	Fucation
MdMRS2-1-F	5′-ACTTCACCTGTCAGCGACTCTGG-3′	
MdMRS2-1-R	5′-CTCGCCCTGCTTGTTCTTGATACC-3′	
MdMRS2-2-F	5′-TCTCCGCCGCAGCCGATTC-3′	
MdMRS2-2-R	5′-CGCCGCCAGCAACAACCG-3′	
MdMRS2-3-F	5‘-ATGGAGGACCTCAAAGAGAGGC-3’	
MdMRS2-3-R	5‘-TAGTGGCATCAGTCTTCGATACTTG-3’	
MdMRS2-4-F	5′-TTGGACGTGGACAAGTATGGCATC-3′	
MdMRS2-4-R	5′-GCGGAAGGGTAAGAGAGCAAAGG-3′	
MdMRS2-5-F	5′-CTCCTCTGCCCACCAACAATGC-3′	qRT-PCR
MdMRS2-5-R	5′-CCCTTCACGCCACCAACAACC-3′	
MdMRS2-6-F	5′-GTGGTGGCGGTGAAGAAGAAGTC-3′	
MdMRS2-6-R	5′-GCGTGAATGTGAACTCTGTGCATG-3′	
MdMRS2-7-F	5′-GCAGTATGTGGTGGAGCTTCAGAG-3′	
MdMRS2-7-R	5′-CGACTCAAGCCAGAACCATCAGAC-3′	
MdGAPDH-F	5′-GAGCTCGCAGGTATCCTTTCT-3′	
MdGAPDH-R	5′-TACCAAGCAATGACCTTGACC-3′	
MdMRS2-3-GFP-F	5‘-ATGGAGGACCTCAAAGAGAGGC-3’	
MdMRS2-3-GFP-R	5‘-TAGTGGCATCAGTCTTCGATACTTG-3’	Clone
