# Supplementary figures and images for: Identification of MRS2 Gene Family and Expression Analysis in Response to Magnesium Treatment in Malus domestica
Source: Plants (Basel). 2025 May 30;14(11):1672. doi: 10.3390/plants14111672 (PMC12157029; doi:10.3390/plants14111672)

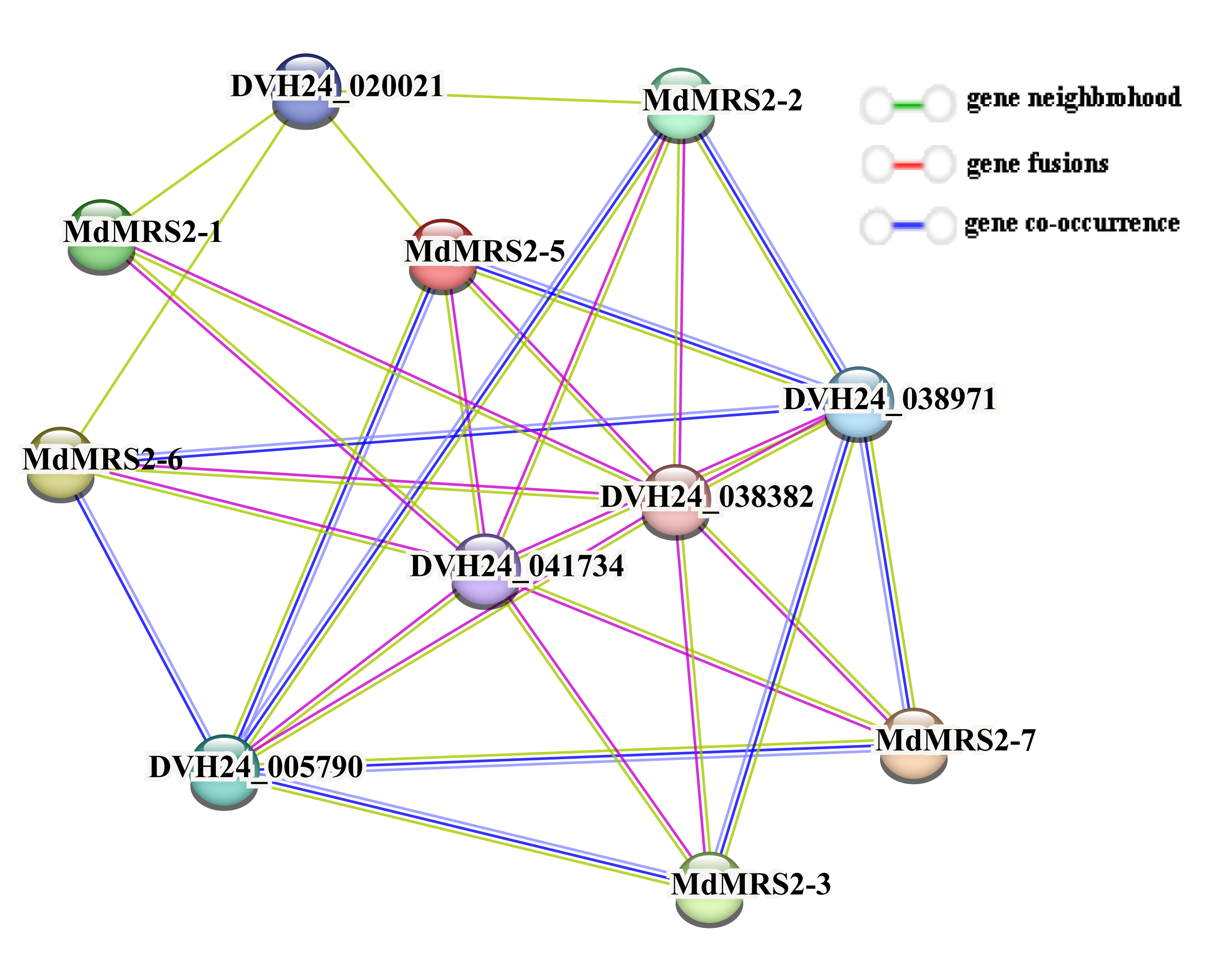

Supplement: Supplementary file 1 [file plants-14-01672-s001.zip › Figure.s1.tif]
